# Does Orexin B-Binding Receptor 2 for Orexins Regulate Testicular and Epididymal Functions in Normal and Cryptorchid Dogs?

**DOI:** 10.3389/fvets.2022.880022

**Published:** 2022-07-12

**Authors:** Caterina Squillacioti, Alessandra Pelagalli, Loredana Assisi, Anna Costagliola, Luc Van Nassauw, Nicola Mirabella, Giovanna Liguori

**Affiliations:** ^1^Laboratory of Anatomy, Department of Veterinary Medicine and Animal Productions, University of Napoli Federico II, Naples, Italy; ^2^Department of Advanced Biomedical Sciences, University of Napoli Federico II, Naples, Italy; ^3^Institute of Biostructures and Bioimages (IBB), National Research Council (CNR), Naples, Italy; ^4^Department of Biology, University of Napoli Federico II, Naples, Italy; ^5^Laboratory of Human Anatomy and Embryology, Department ASTARC, Faculty of Medicine and Health Sciences, University of Antwerp, Antwerp, Belgium; ^6^Department of Prevention, ASL FG, Foggia, Italy

**Keywords:** orexins, testis, epididymis, cryptorchidism, steroidogenesis, dog

## Abstract

Orexins A (OXA) and B (OXB) and the receptors 1 (OX1R) and 2 (OX2R) for orexins are hypothalamic peptides found in several mammalian organs and participated to the control of a wide assortment of physiological and pathological functions. The distribution of OXA and OX1R has been extensively studied in the male gonad of mammals. Here, we examined the expression and localization of OXB and OX2R as well as their possible involvement in the regulation of testicular and epididymal functions, in healthy and cryptorchid dogs, employing some techniques such as immunohistochemistry, Western blotting, and real-time RT-PCR. In *vitro tests* were also carried out for evaluating the steroidogenic effect of OXB. OXB and OX2R were expressed in spermatocytes, spermatids, and Leydig cells in normal testis. Their localization was restricted to Sertoli and Leydig cells in cryptorchid conditions. OXB was found to be localized in all tracts of both normal and cryptorchid epididymis, whereas OX2R was found only in the caput. Because the small molecular weight of the peptides OXA and OXB, the expression of their precursor prepro-orexin (*PPO*), *OX1R*, and *OX2R* proteins and mRNAs were investigated by means of Western blot and real-time RT-PCR analyses, respectively, in all tested groups of. In particular, the mRNA level expression of all three genes was higher in cryptorchid dogs than in normal ones. In *vitro tests* demonstrated that OXB—by binding OX2R—is not involved in testicular steroidogenic processes. Therefore, the findings of this study might be the basis for further functional and molecular studies addressing the possible biochemical effects of OXB and OX2R in normal and pathological conditions of the male reproductive system.

## Introduction

Orexins A (OXA) and B (OXB) represent peptides of hypothalamic origin that derived from a common precursor known as prepro-orexin (PPO) (1, 2). The physiological effects of the before mentioned peptides are mediated by the interaction with two G-coupled receptors—orexin 1 (OX1R) and 2 (OX2R)—for orexins, in which, OX1R is specific for OXA while OX2R has the same binding affinity for both peptides. Apart from their implication in regulating of food consumption and of spontaneous physical activity, recent literature has demonstrated a preponderant role, especially for OXA, in the modulation of male reproductive actions (1, 2).

In the male genital tract, PPO, OXA, and OX1R were found in the testis and epididymis of different animal species, dogs included (3–13), and in the human prostate (14). In contrast, the distribution of OXB and OX2R has been described in the testis of rats and alpaca (15, 16) and rat epididymis (17), and *OX2R* mRNAs were detected in several male genitalia (18) and in the prostate of humans (19).

Notoriously, OXA—by binding OX1R—has been demonstrated to have a steroidogenic effect in the testes of rats (5), alpaca (8), and normal and cryptorchid dogs (9). The mechanisms as to how OXA-binding OX1R regulates testicular steroidogenesis has been well-demonstrated in adult mice by Joshi et al. (12). These findings confirmed our recent papers in dogs, where OXA was demonstrated to significantly decrease basal 17βE secretion *via* a marked reduction in the aromatase (ARO) enzymic activity in normal and cryptorchid gonads, respectively (6). As it is known, ARO is responsible for the aromatization of androgens into estrogens (20). In contrast, OXB was demonstrated to not affect steroidogenesis (15, 16), thus its role remains unclear. The regulation of male fertility and differentiation depends on the testicular descent from the abdomen to the scrotum (21). In this regard, cryptorchidism is a testicular dysgenesis has been described as the failure of one (unilateral) or both (bilateral) testes and the relative spermatic ducts to migrate into the scrotum (22–24), that is found particularly in dogs (involving, to a greater extent, the right gonad) (25–27), stallions (21, 23, 28), and humans (29). The abnormal localization of the retained gonad and spermatic ducts causes impaired fertility and increases carcinogenesis associated to Sertoli cell tumors and seminomas (21). Histological alterations of testis and epididymis in normal and cryptorchid dogs have been previously described (30).

Therefore, in order to deepen our knowledge on this topic, we elucidated the distribution of OXB and OX2R in the testis and epididymis of normal and cryptorchid dogs by means of immunohistochemistry. In addition, Western blotting analysis of PPO and OX2R, and Real-time RT-PCR analysis to establish mRNA levels of *PPO*, and *OX2R*, the latter in comparison with *OX1R*, were carried out in the testis and epididymis of normal and cryptorchid animals. A possible functional relationship between OXB and ARO was studied focusing on Oestradiol 17β (17βE) synthesis from Testosterone (T), using *in vitro* cultured testicular slices.

## Materials and Methods

### Animals and Tissues

The experimental procedures were conducted on 20 adult male mixed breed dogs of different body weights and ages which were classified into two groups: control group (*n* = 10, average weight 19.8 ± 2.7 kg, average age = 4.8 ± 1.91 years) and dogs with cryptorchidism (unilateral abdominal: *n* = 10, average weight 18.0 ± 2.0 kg, average age = 4.2 ± 1.64 years). All dogs were presented to the Veterinary clinic of the University Federico II, Naples, Italy, for orchiectomy. Written informed consent has been provided by the owners to enroll their dogs in the study. Testes and epidydimides were removed surgically following the verbal consent of the owners regarding the surgical and general sample collection procedures.

Selected animals were not previously involved in any clinical trials or treatments. All handling and experimental procedures were in compliance with and approved by the Ethical Animal Care and Use Committee of the University of Naples Federico II, Department of Veterinary Medicine and Animal Production, Naples, Italy (no. 0,050,377). Tissue specimens were fractionated into two groups: (1) normal testis and epididymis and (2) cryptorchid testis and epididymis. Epididymides were divided into three segments: (1) caput, (2) corpus, and (3) cauda. In detail, the epididymis was first dissected from the testis and then, the caput and cauda epididymal segments were cut from the entire segment according to the macroscopic exam of the organ. Therefore, the remaining segment represented the corpus. To refine the tissue, their left and right margins were isolated. Afterwards, fixation (Bouin) of tissues was rapidly performed for 12–24 h (*n* = 5 animals for each group). Moreover, tissues used for Western blotting, RT-PCR, and *in vitro* tests (*n* = 5 animals for each group) were collected and immediately frozen in dry ice and stored at −80°C until use.

### Immunohistochemistry

Tissues collected were processed as previously described (9). Briefly: tissue sections (7 μm thick) were deparaffinized by immersion in xylene for 10 min (2x) and rehydrated by passing through a series of descending ethanol concentrations. Then, sections were immersed in 10 mM citrate buffer (pH 6.0), exposed to high temperature in microwave oven (700 W) for 5 min (2x) in order to unmask the antibody binding site of the proteins, and incubated in 3% H_2_O_2_ in a humid chamber for 20 min. Any non-specific antibody reaction was blocked by incubating the sections in normal goat serum (S1000, Vector Laboratories, Burlingame, CA, USA) (1:66 dilution in phosphate-buffered saline PBS 7.2%) for 30 min. In particular, the blocking step is essential for preventing non-specific binding of antibodies or other reagents to the tissue. Even if the antibody has high specificity toward the target, intermolecular forces can promote non-specific binding to other molecules. Following blocking, slides were washed with PBS or 5 min (3x) and incubated with mouse monoclonal anti-human OXB (MAB734, R&D System, Abingdon, UK, that shares 89,313% identity with dog: UniProt.Org) and rabbit polyclonal anti-rat OX2R antibody (AB3094, Millipore, Billerica, MA, USA, that shares 91,75% identity with dog: UniProt.Org). After 3x PBS washes, the sections were treated with a secondary antibody, ultra-polymer goat anti-rabbit/mouse IgG (ImmunoReagents, Raleigh, NC, USA) conjugated with a peroxidase polymer backbone (1:4) and incubated for 30 min in a humid chamber. After washing with PBS, the cells were incubated with 3,3′-diaminobenzidine tetrahydrochloride (DAB) (SK-4100, Vector Laboratories, Burlingame, CA, USA) and dehydrated in ascending ethanol concentrations followed by xylene. Next, counterstaining was performed by using hematoxylin in order to identify the testicular cytotypes and clearly visualize the specific localization of immunoreactions. The negative controls (data not shown) included omission of primary antibody as previously described (17).

Observation of the immunoreactions and image documentation was performed by three different blind observers on a total of 600 sections, using a Leica DM 6B light microscope and SFC7000T digital camera.

### Western Blotting

Canine specimens were collected for Western blotting, as described elsewhere (9). Briefly, the tissues were homogenized in ice-cold RIPA buffer (50 mM Tris-Cl pH 7.4, 150 mM NaCl, 10% glycerol, 0.1% SDS, 1% Triton X-100, 0.5% deoxycholate) complemented with 1x protease cocktail inhibitors and centrifuged at 14,000 rpm for 15 min, at 4°C. After centrifugation, the supernatant was recovered and protein was determined by the Bradford assay (Bio-Rad Laboratories Inc., Hercules, CA, USA). Equal protein contents (30 μg) resuspended in Laemmli buffer were boiled for 4 min and loaded on a 4–20% Mini-PROTEAN, TGX Stain-Free Precast Electrophoresis Gel (Bio-Rad Laboratories, Inc., Hercules, CA, USA). After electrophoresis, proteins were transferred into membranes by the use of a Mini Trans-Blot apparatus (tank/wet transfer method) (Bio-Rad Laboratories Inc., Hercules, CA, USA), and this procedure was assessed using a ChemiDoc molecular imager (Bio- Rad Laboratories, Inc., Hercules, CA, USA). With the purpose of blocking proteins transferred into membrane, it was held in blocking solution containing 5% non-fat milk diluted in TBS-T buffer (1.5 M NaCl, 200 mM Tris-HCl, and 0.1% Tween-20, pH 7.2) at room temperature. At the end of 1-h blocking and after washing in TBS-T, the blot was incubated overnight at 4°C with the primary antibodies; the small molecular weight of the peptides OXA and OXB, did not allowed us to highlighted them in the extract, thus, their precursor PPO was researched (9) [rabbit polyclonal anti-PPO antibody (1:500 dilution), AB3096-Millipore, Temecula, CA, USA] and OX2R [rabbit polyclonal anti-OX2R (1:500 dilution), ab3094, Millipore, Billerica, MA, USA]. Then, three washes with TBS-T for 10 min were performed and the blot was subjected to incubation with a goat anti-rabbit secondary antibody conjugated with horseradish peroxidase (HRP) (ImmunoReagents, Raleigh, NC, USA; 1:1000 dilution) for 1 h at room temperature were performed. After the last three washes with TBS-T, ECL (Bio-Rad Laboratories, Inc., Hercules, CA, USA) was adopted for visualizing the proteins, and an image was captured by the ChemiDoc molecular imager (Bio-Rad Laboratories, Inc., Hercules, CA, USA). The standard molecular weight marker used was Precision Plus Protein^TM^ All Blue Prestained Protein Standards (10–250 KdA, #1610373, Bio-Rad Laboratories, Inc., Hercules, CA, USA). Whole rat brain homogenate was considered as positive control (data not shown).

### Real-Time RT-PCR

Total RNA extraction and cDNA synthesis were made as reported in literature (31). Total RNA was obtained homogenizing tissues in ice-cold TRIzol reagent using an Ultra-Turrax homogenizer (cat. n.: 15596026, invitrogen, California, USA). After extraction, the RNA was dissolved in RNAase-free diethyl dicarbonate (DEPC) water. After this step, the RNA quantification was performed using an Eppendorf BioPhotometer (Eppendorf AG, Basel, Switzerland). One microgram of total RNA was retrotranscribed using High-Capacity cDNA Reverse Transcription Kits (Applied Biosystems, Carlsbad, CA, USA), according to the manufacturer's instructions, using random hexamers as the primers. The mRNA transcript profiles for these genes in the dog species was studied with Quantitative real-time RT-PCR, TaqMan probes. The following TaqMan gene expression assays (Applied Biosystems Applied Biosystems, Carlsbad, CA, USA) were used: *HCRTR2* (Cf02623700_m1); *HCRT* (Cf02695831_s1); *HCRTR1* (Cf02695834_u1); *GAPDH* (Cf04419463_gH) (Table 1). The real-time PCR reactions contained 1 μL of cDNA (50 ng/well) and 19 μL TaqMan® Fast Advanced Master Mix Containing primers and TaqMan probes specific for these genes. The PCR conditions were here described: 50°C for 2 min and 94°C for 10 min, followed by 40 cycles of 94°C for 15 s and 60°C for 1 min. As an active endogenous reference in order to normalize the quantification of the mRNA target, the *GAPDH* gene was amplified in separate tubes under the same conditions to serve. An ABIPRISM 7300 Sequence Detection System (Applied Biosystem, Foster City, California, CA, USA) was adopted for Real-time, and data for TaqMan PCR amplicons were evaluated with the ABI 7,300 System SDS software. The relative expression for all genital segments was calibrated by means of the delta-delta Ct method (2DDCt), as reported previously (32). *PPO, OX1R* and *OX2R* expression levels between the different samples (testis, caput epididymis, corpus epididymis, and cauda epididymis) were normalized by using *GAPDH* expression in normal and cryptorchid dogs.

**Table 1:** List of Taqman assays for real time RT-PCR.

**Gene name**	**Assay ID**	**Amplicon length (bp)**
*HCRTR2*	Cf02623700_m1	83
*HCRT*	Cf02695831_s1	65
*HCRTR1*	Cf02695834_u1	86
*GAPDH*	Cf04419463_gH	54

*Hypocretin (HCRT); Hypocretin Receptor1 (HCRTR1); Hypocretin Receptor 2 (HCRTR2); Glyceraldehyde-3-Phosphate Dehydrogenase (GAPDH)*.

### *In vitro* Determination of Testosterone (T) and 17βEstrogen (17βE) Levels

Each group of tissues was cut into pieces (250 ± 7 mg) of 400 μm thick and allocated in multi-well plates (2 slices/well) and treated with 2 mL Krebs–Ringer bicarbonate buffer (KREB), [10 mM glucose, 100 μM bacitracin, 0.1% ascorbic acid, 0.1% bovine serum albumin (BSA), pH 7.4], for 60 min at 37°C in a 95% O2/5% CO2 atmosphere with constant shaking at 60 cycles/min. The samples were then treated with a fresh solution of OXB peptide (1 nM) (003-32, Phoenix Pharmaceutical, Karlsruhe, Germany) or OXB + OX2R antagonist (TCS OX2 29, Tocris Bioscience, Bristol, UK) in fresh KREB buffer at 37°C for 12 h. Samples for positive and negative controls were treated with 1 nM Luteinizing hormone (LH) of sheep pituitary (L5269, Sigma Chemical, MO, USA), and medium (KREB) alone, respectively. In the next phase of the experiment, the samples were diluted with ethyl ether (1:10) and the supernatant (the ether phase) was collected into a new test tube. The ether base extraction was repeated 2–3 times by centrifugation at 3,000 × *g* for 10 min. The pooled ether extracts were evaporated at room temperature, and the residue was dissolved in a 0.5 mL sodium phosphate buffer 0.05 M (pH 7.5). Finally, 50 μL of the diluted samples was distributed into a 96-well plate, incubated with EIA reagents and substrates consistent with the manufacturer's instructions and evaluate by a Biotech photometer at a wavelength of 545 nm (EIAgen Testosterone Kit, Adaltis, Bologna, Italy; estradiol DKO003, Diametra, Perugia, Italy).

### Aromatase Activity Assay

The *in vitro* conversion rate of T to 17βE assayed in fresh tissues was used to measure ARO activity. One specimen (250 ± 7 mg) of male gonad samples was distributed in each well of a multi-well plate. Then, suspension of the tissue was treated with the above-reported substances followed by the addition of T (35 μM) dissolved in 100 μl of NADPH solution (3 mg/mL) and treated for 24 h at 37°C. Afterwards, the hormone was extracted with ether and left to dry at room temperature. 17βE determination was carried out using the residues of this extract through an ELISA immunoassay (EIA) kit as reported previously (6). The results were indicated as the 17βE content produced per gram of tissue/per hour.

### Data Analysis

All data relative to Real-time RT-PCR and to the *in vitro* tests are presented as mean ± S.D. For the Real-Time RT-PCR, variance analysis (ANOVA) for unpaired data and Tukey's HDS test for independent samples were assessed for analyzing the significance of differences in the relative contents of *PPO, OX1R*, and *OX2R* mRNA between the different samples of both healthy and cryptorchid animals. As well, statistically significant differences in *PPO, OX1R*, and *OX2R* mRNA levels between the calibrator (healthy segments) and retained counterparts were investigated using Student's *t*-tests. The results acquired from *in vitro* tests were measured by analysis of variance (ANOVA) followed by Duncan's test for multi-group comparison and Student's *t*-test for between-group comparison. All the experiments relative to the indicated procedures were carried out in triplicate. The level of significance was attributed at *p* < 0.01 and *p* < 0.05.

## Results

### Immunohistochemical Analysis of OXB and OX2R in Testes and Epididymides From Normal and Cryptorchid Dogs

**Figure 1:**
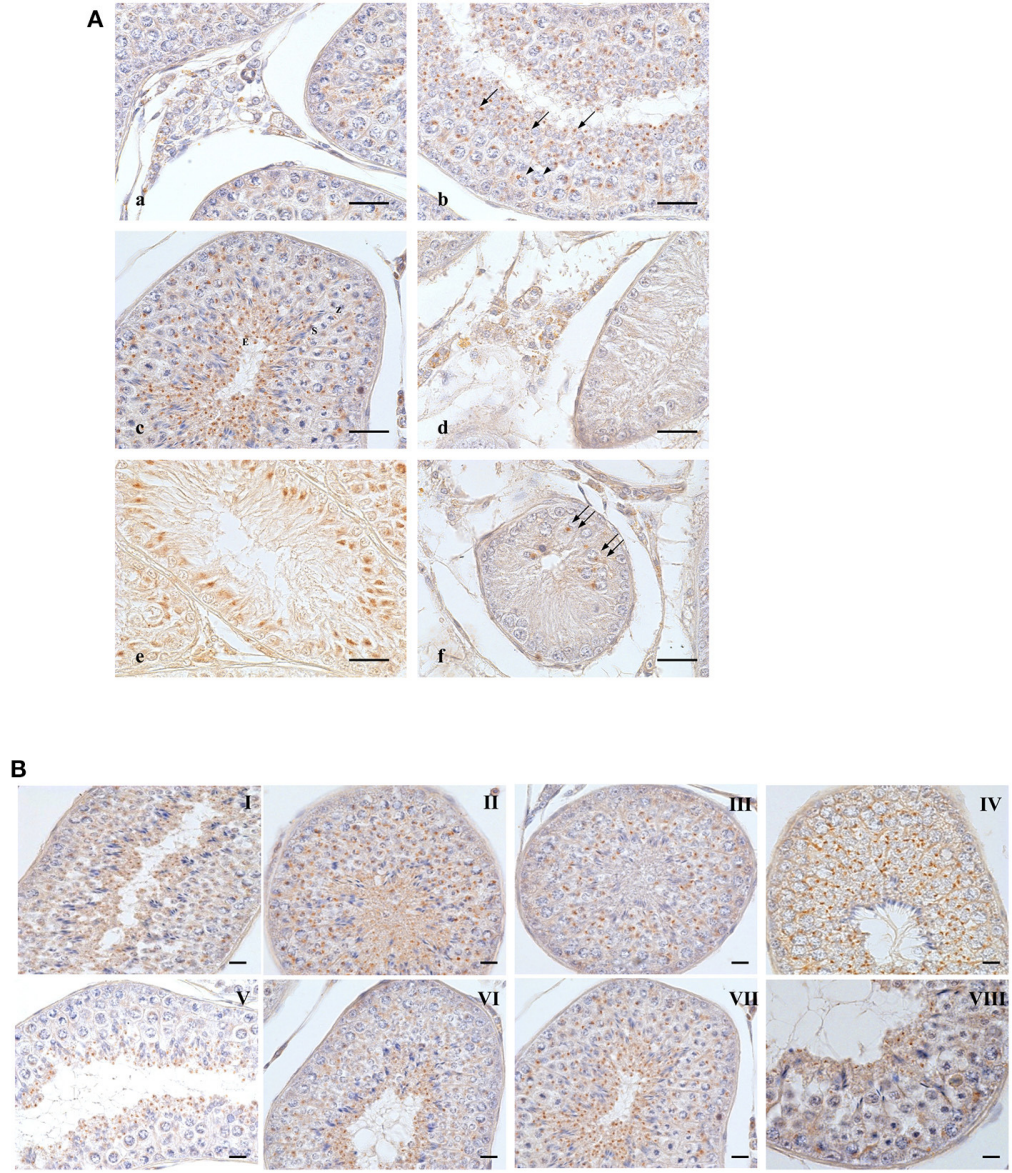
OXB-IR in normal and cryptorchid canine testis. **(A)**: Group of Leydig cells labeled with fine granular stains dispersed through the cytoplasm (a); pachytenes (b, arrowheads) and zygotenes (c,Z) are stained with single round deep stain located close to the nuclear membrane; smaller roundish punctiform stain in round (b, arrows), secondary spermatocytes (c,S) and elongated spermatids (c,E). In spermatids, the immunoreactive substances are always found in the posterior portion of cytoplasm behind the nucleus, toward the luminal side of the seminiferous tubule. In cryptorchid testis, fine granular stain is distributed in the cytoplasm of Leydig cells with intermingled unstained cells (d); profound immunoreactions are observed junctional area of Sertoli cells (e). Staining in gonocytes is described as a deep round granule located in perinuclear area of cytoplasm (f). **(B)**: distribution OXB-IR along the germ developing cycle. Bars: 20 μm.

**Figure 2:**
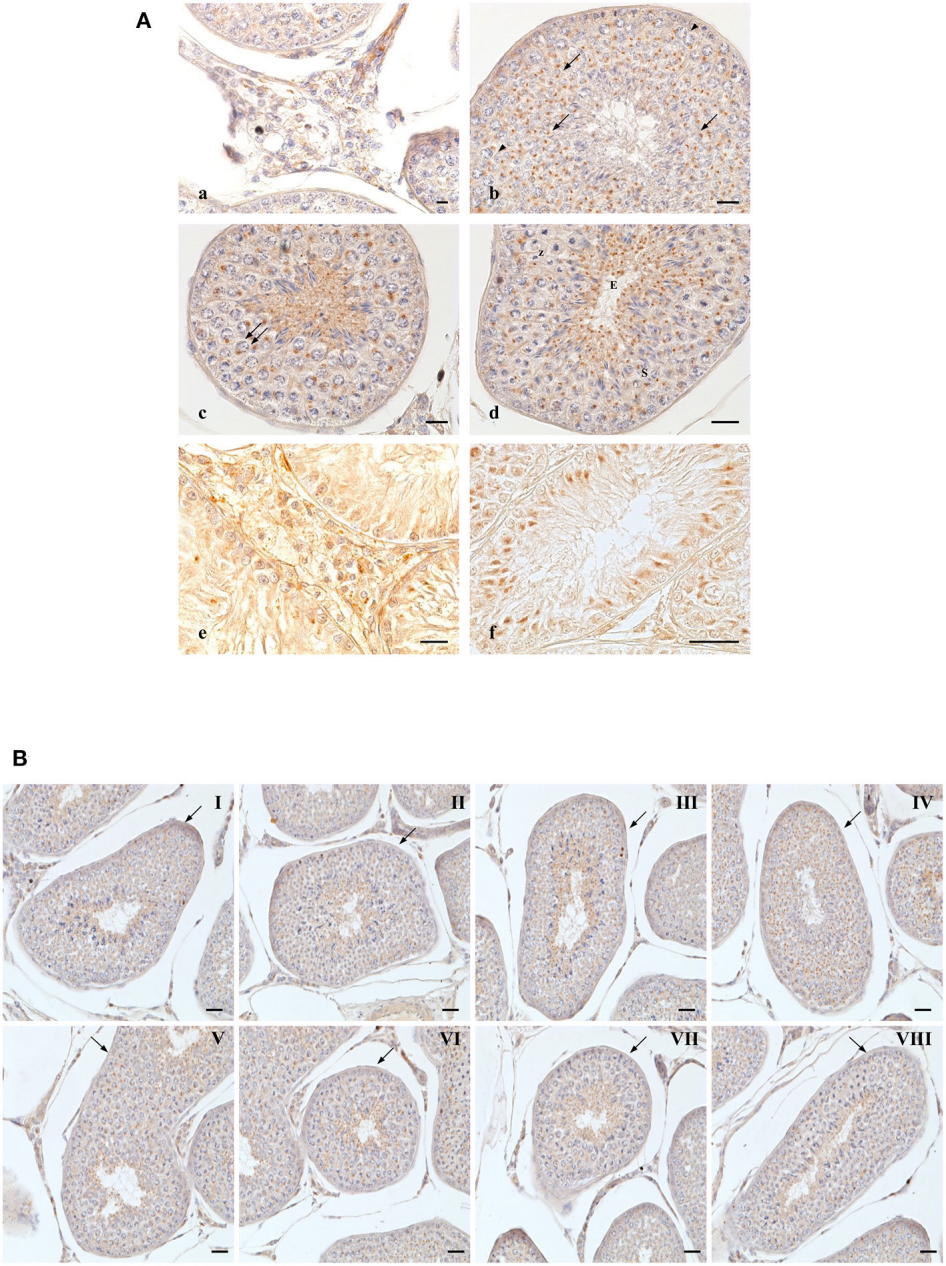
OX2R-IR in normal and cryptorchid canine testis. **(A)** Group of Leydig cells labeled with micro-granular material through the entire cytoplasm (a); deeply stained single round granular structure located in perinuclear area of pachytene (b, arrowheads), diplotene (c, double arrow), zygotene (d,Z). Relatively smaller compact punctiform materials are observed in secondary spermatocytes (d,S); round (b, arrows) and elongated (d,E). In cryptorchid dog testes. Leydig cells are stained with finer granular substances diffused through the entire cytoplasm (e); intensive immunoreactions are observed the Sertoli cells, particularly in basal and apical regions (f). Bars: 20 μm. **(B)** OX2R-IR in normal dog testes along the entire germ developing cycle. Bars: 30 μm.

**Figure 3:**
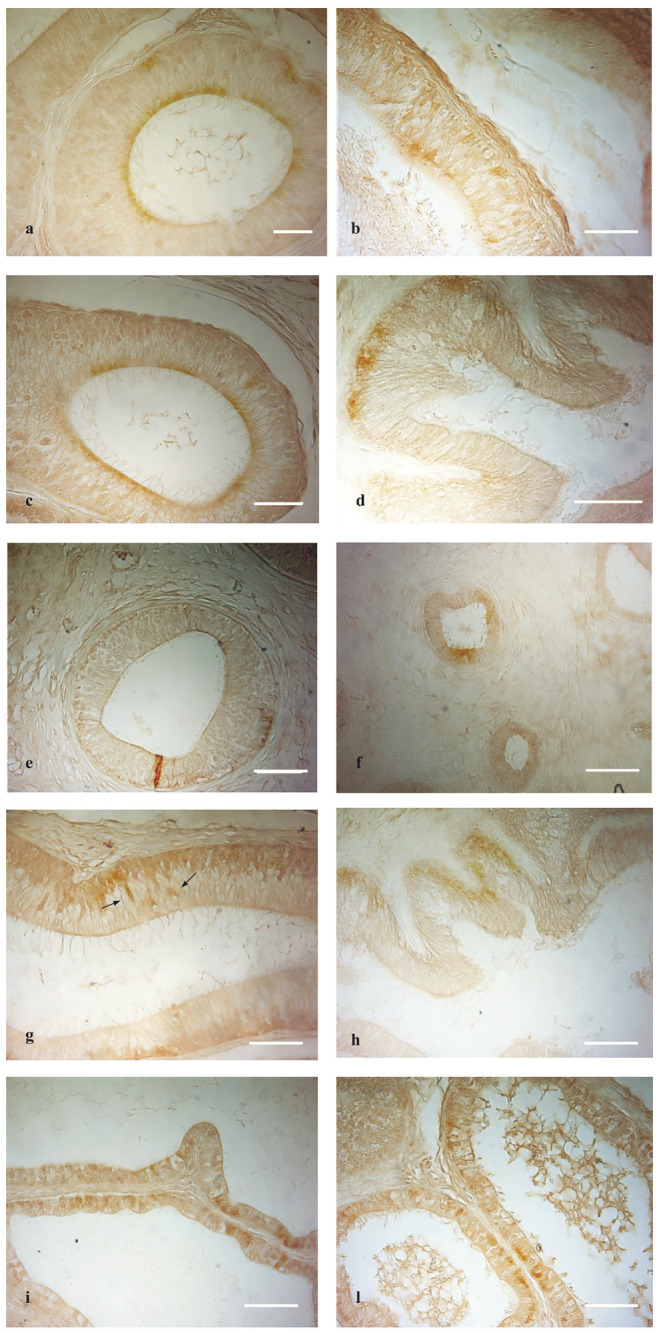
OXB- and OX2R-IR in normal and cryptorchid epididymis: **(a,b)** OXB-IR was found in the apical and basal portion of the principal cells of the normal caput and corpus epididymis. **(c,f)** In the cauda of normal and cryptorchid epididymis, in which the immunoreactivity was found in the apical portion of principal cells in both normal and cryptorchid cauda epididymis. **(d)** In cryptorchid caput only the basal portion of the principal cells are positive. **(e)** In cryptorchid corpus, rare cells show immunoreactivity. **(g,h)** OX2R-IR was described in the basal portion of the principal cells. Moreover, OX2R-IR was detected in narrow cells [**(g)**, arrows]. **(i,l)** In the normal dogs, OXB and OX2R were distributed in the epithelium lining the epididymal efferent ductules, respectively. Bar: 10 μm.

The results of the immunohistochemical analyses of OXB and OX2R in the testis and different epididymal tracts of normal and cryptorchid dogs are displayed in Figures 1–3, respectively. OXB-immunoreactivity (IR) was described in both the interstitial and tubular cytotypes of the normal and retained male gonad (Figure 1A).

In the normal dog testis, a group of Leydig cells was stained with fine granules dispersed throughout the entire cytoplasm. The positive cells were intermingled among the negative ones (Figure 1A). In the seminiferous tubules, round shaped OXB-positive granules were most frequently observed in the cytoplasm, at the perinuclear region of pachytene spermatocytes (Figure 1b arrowheads). Zygotene spermatocytes (at stage VIII) showed a staining pattern similar to that of pachytene ones (Figure 1c,Z). A similar positive material was observed in secondary spermatocytes (Figure 1c,S). In spermatids, a cluster of small positive-granules changed from round shape (Figure 1b, single arrow) to slightly oval punctiform shape during the differentiation from round or immature spermatids to elongated or mature ones. During these steps, the positivities moved toward the lumen of the seminiferous tubules (Figure 1c,E). The change in staining pattern between round and elongated spermatids most likely corresponds to acrosomal development.

In cryptorchid testis, OXB-IR was observed in Leydig (Figure 1d) and Sertoli (Figure 1e) cells and rarely detected in early gonocytes (Figure 1f, double arrows).

In normal animals, OX2R-IR was evidenced in cells located in the interstitium and in seminiferous tubules (Figure 2A). In Leydig cells, positive-fine granules were distributed within the cytoplasm (Figure 2a), while in pachytene spermatocytes, roundish condensed positive-granule were found close to the nuclear membrane (Figure 2b, arrowheads). Similar staining features were detected in diplotene (Figure 2c, double arrow) and zygotene (Figure 2d,Z) spermatocytes and in secondary spermatocytes (Figure 2d,S). In spermatids, as previously seen for OXB, OX2R-positive-material was detected throughout the spermatid maturation from round (Figure 2b, arrows) to elongated (Figure 2d,E) spermatids.

In the cryptorchid testis, immunopositive material was predominantly observed in Leydig (Figure 2e) and Sertoli (Figure 2f) cells. Interestingly, intense immunopositivity was detected in the most basal and apical cytoplasmic portions of Sertoli cells (Figure 2f).

OXB- and OX2R-IR were found in all stages of the testicular germ developing cycle (Figures 1, 2B) accordingly to Soares et al. (33). The semi-quantitative localization of OXB and OX2R in the male gonad of normal and cryptorchid dogs is summarized in Table 2.

**Table 2:** Localization of OXB and OX2R in the male gonad of normal and cryptorchid dogs.

	**Cytotypes**						
	**Leydig**	**Sertoli**	**Early germ**	**Primary**	**Secondary**	**Round**	**Elongated**
	**cells**	**cells**	**cells**	**spermatocytes**	**spermatocytes**	**spermatids**	**spermatids**
**OXB**
Normal testes	+	–	–	+	+	+	+
Cryptorchid testes	+	+	+	–	–	–	–
**OX2R**
Normal testes	+	–	–	+	+	+	+
Cryptorchid testes	+	+	–	–	–	–	–

*+++ high density; ++ moderate density; + low/mild density; – absence*.

OXB-IR was identified in all segments of the epididymis both in normal and cryptorchid dogs (Figures 3a–f).

In normal animals, OXB-IR was observed in the basal and apical portions of the principal cells of the caput and corpus epididymis (Figures 3a,b). In the cauda region positive reactions were detected in the cytoplasmic apical portion of the principal cells (Figure 3c).

In cryptorchid animals, OXB-IR was observed in the basal portion of principal cells of the caput epididymis (Figure 3d), while in the corpus, rare narrow, intensely stained cells were observed (Figure 3e). In the cauda, OXB-IR was found in the apical and basal portions of the principal cells (Figure 3f).

OX2R-IR was present only in the caput of both the normal and cryptorchid epididymis (Figures 3g,h).

Both in the normal and cryptorchid caput epididymis, positive-material was found to be widely disseminated in the cytoplasmic basal portion of the principal cells (Figures 3g,h). In the normal animals, these cells were intermingled with scattered narrow cells (Figure 3g, arrows). In addition, intense OXB- and OX2R-positive granules defining the entire cytoplasmic profile as well as the basal portion of the efferent ductules epithelium (Figures 3i–l). In the not retained gonad and its relative epididymis of the cryptorchid animals, the distribution of OXB and OX2R was similar to those observed in the normal dogs (data not shown).

The immunolocalization of both the peptides in the normal and cryptorchid epididymal cells is summarized in Table 3.

**Table 3:** Immunohistochemical density of OXB and OX2R IRs in the epididymal efferent ductules and epididymis of normal and cryptorchid dogs.

**Normal**		**OXB**	**OX2R**
**Epididymis**	**Efferent ductules**	**+++**	**+++**
	**Caput**	**++**	**+++**
	**Corpus**	**++**	**–**
	**Cauda**	**+**	**–**
**Cryptorchid**
**Epididymis**	**Efferent ductules**	**+++**	**+++**
	**Caput**	**++**	**+++**
	**Corpus**	**+**	**–**
	**Cauda**	**++**	**–**

*+++ high density; ++ moderate density; + low/mild density; – absence*.

### Western Blot of PPO and OX2R Peptides

**Figure 4:**
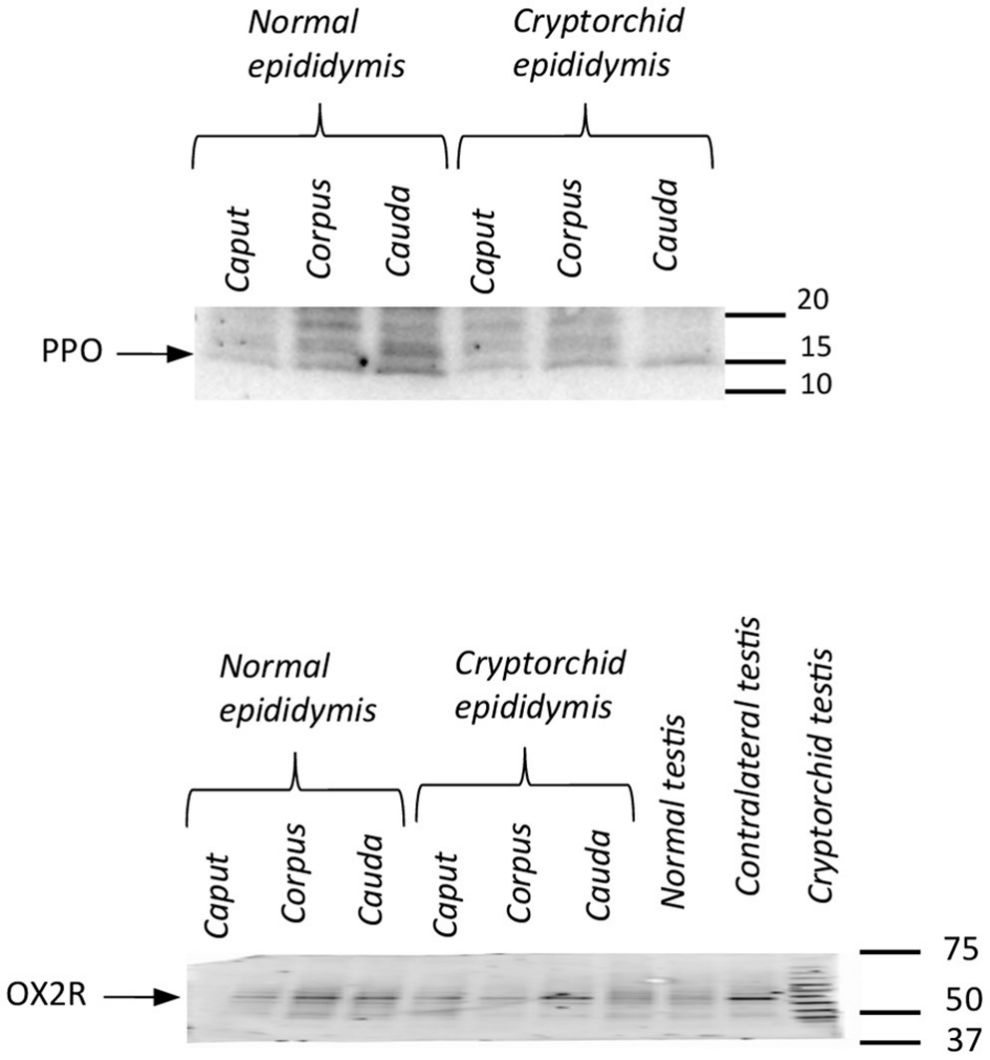
Western blotting of PPO and OX2R in normal and cryptorchid dogs. Representative Western blot detection of OX2R **(A)** and PPO **(B)** in dog tissues. Data are representative of three independent experiments.

The tissue extracts from the testis and epididymis of the normal and cryptorchid dogs reacted with specific antibodies. Anti-PPO antibody recognized a major protein band at approximately 16 kDa and anti-OX2R antibody recognized a major protein band at ~50 kDa (Figure 4).

### mRNA Expression of *PPO, OX1R*, and *OX2R* in the Testis and Epididymis of Normal and Cryptorchid Dogs by Real-Time RT-PCR

**Figure 5:**
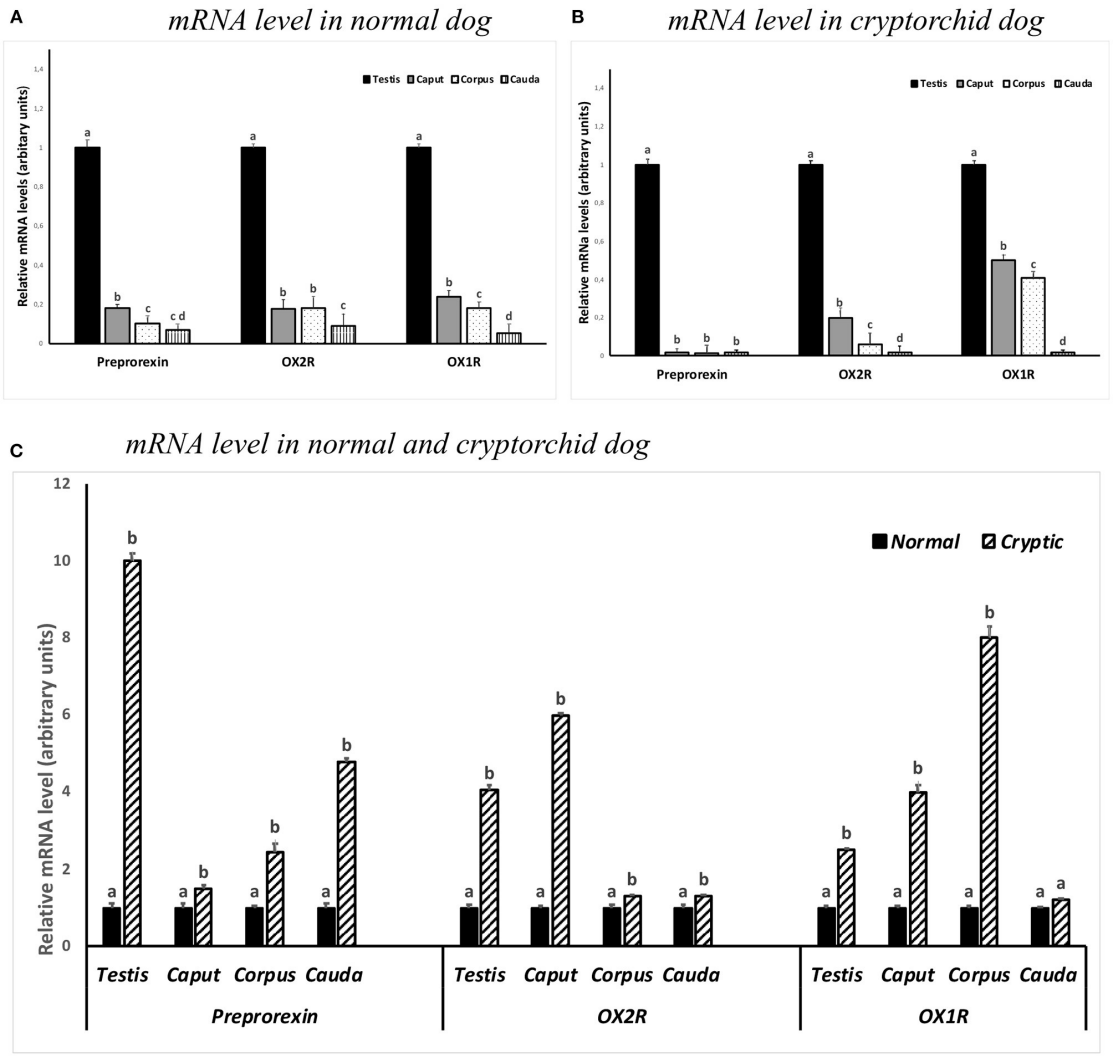
*PPO, OX1R* and *OX2R* mRNA levels in the normal **(A,C)** and cryptorchid dogs **(B,C)**. **(A)** The calibrator is the normal testis; **(B)** the calibrator is cryptorchid testis; **(C)** the calibrator is the segment of the normal dog. Each value is expressed as the mean ± standard deviation (SD) obtained from three independent experiments. Different letters depict differences between the examined groups (*n*=*5; p* < 0.05).

Real-time RT-PCR analysis was performed to determine the expression of *PPO*-, *OX1R*-, and *OX2R*-mRNAs in the testis and in the different segments of the epididymis from normal and cryptorchid dogs. The expression of *OX2R*-mRNA level was also compared with that of *OX1R*-mRNA, not studied previously. As displayed in Figure 5A, in the healthy animals, all three genes were found in the testis and epididymal portions. The mRNA levels decreased along the genital tract from the testis to the cauda of the epididymis.

In the cryptorchid dogs, a similar trend level in the mRNA expression for *OX1R*-, and *OX2R*-mRNA was observed. In contrast, mRNA expression of *PPO* was considerably low in all tracts (Figure 5B).

In particular, an increase in the *OX2R* was particularly high in the testis and in the caput epididymis of cryptorchid animals when compared with the normal samples, as well as an increased *OX1R*-mRNA level in the epididymal corpus of cryptorchid dogs was found when compared with the normals ones (Figure 5C).

### *In vitro* T and 17βE Determination by Elisa Immune Essay

**Figure 6:**
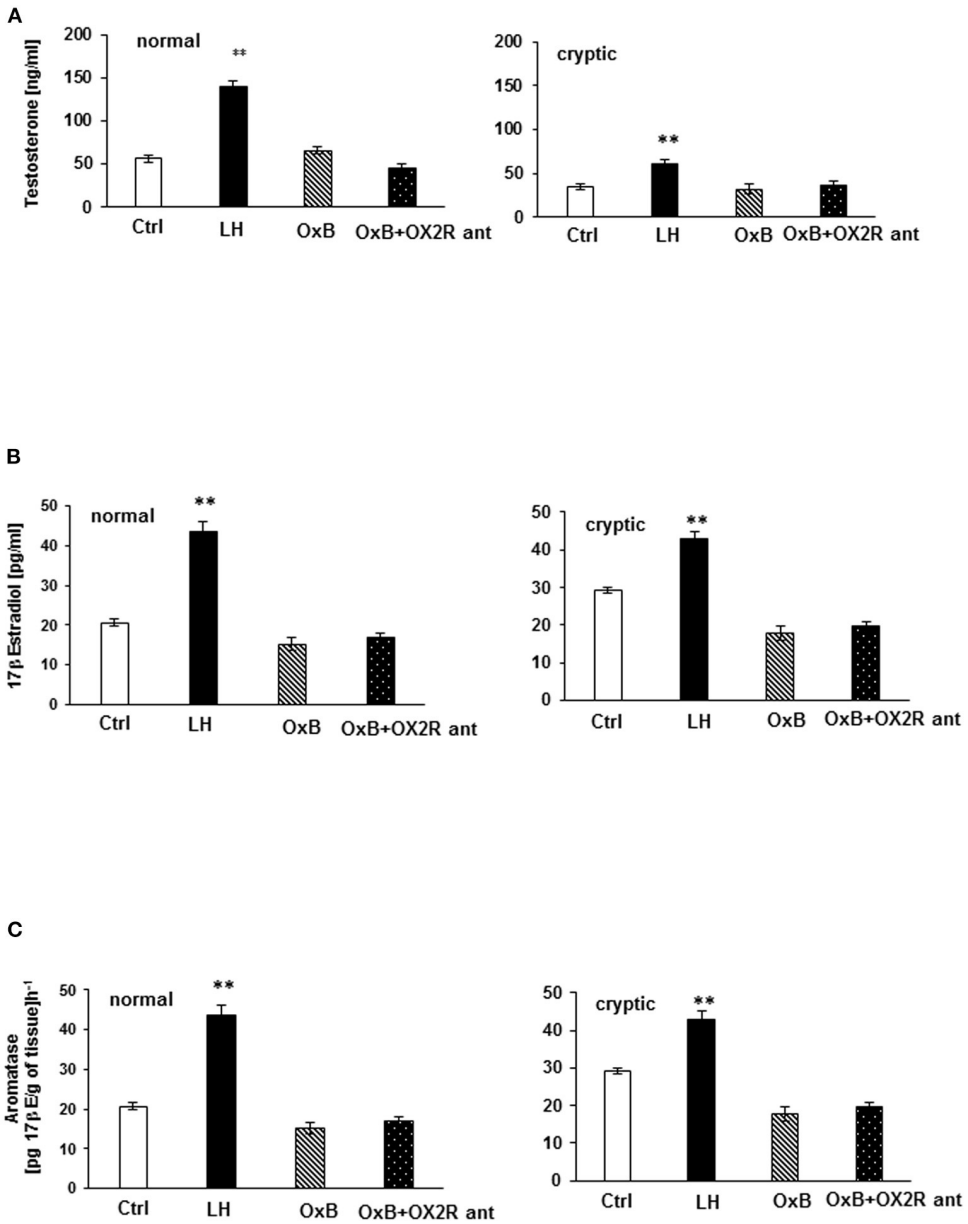
*In vitro* testosterone (T) and 17β Estrogen (17βE) secretion **(A,B)** and aromatase (ARO) activity evaluation in normal and cryptorchid testis of dogs. **(A)** represents T secretion where data are presented in mean ± SD. **P < 0.01 *vs*. corresponding controls. **(B)** represents 17βE secretion, where data are expressed as the mean ± SD; *n* = *5;* *p < 0.05, **p < 0.01 vs. corresponding controls. **(C)** represents ARO activity values are expressed as 17β-estradiol product per gram of tissue per hour. Data are presented as the mean ± SD. **p < 0.01 vs. corresponding controls. All the experiments were conducted after 24 h incubation with LH, OXB, and/or OXB with OX2R antagonist. One-way ANOVA for multi-comparison test was applied for comparison of concentration of the hormone in each test group. The control (CTR) was the tissue alone. “OXB + OX2R ant” stand for groups incubated with OXB and OX2R antagonist.

An *in vitro* test coupled with EIA was applied for the evaluation of the steroidogenic effect of OXB and OX2R selective antagonists in dog testis. T and 17βE secretion levels were measured and analyzed from normal and cryptic canine testicular slides. It was found that neither OXB nor OX2R selective antagonist affected T and 17βE production in all tissue groups. No statistical differences in T and 17βE concentration were found between the treated and control groups. However, T and 17βE concentrations significantly increased in LH-treated groups. In contrast, LH significantly strengthened basal T (*p* < 0.01) and basal 17βE (*p* < 0.01) secretions in the tissues from all tested groups (Figures 6A,B).

In normal and cryptorchid dogs, ARO activity was assessed indirectly by measuring the 17βE concentration of the tissue after 24 h incubation with OXB peptide with or without the OX2R antagonist in the presence of T and LH, as substrates, respectively (35 μM). In this experiment, ARO activity was found roughly higher in cryptorchid testis compared to normal testis as a consequence of lower T production (Figure 6C). As usual, LH significantly increased the ARO activity in all tissue (*p* < 0.01). OXB peptide and OX2R antagonist, however, did not affect ARO activity in all cases.

## Discussion

The current research focused the localization of OXB and OX2R in different cytotypes of normal, and cryptorchid canine testes and epididymides. Immunoreactions for both peptides were detected in the different compartments of the testes in all tissue groups.

The extensive expression of OXB and OX2R in multiple cytotypes of testes strongly suggests a potential role in spermatogenesis and endocrine functions of mammalian testis—most probably acting through autocrine and paracrine pathways (34). The immunolocalization pattern of both the peptides in normal dog testis closely resembles previous findings in rats and alpaca (15, 16). In the cryptorchid male gonad, the localization of OXB and OX2R is restricted to Leydig and Sertoli cells. That might be attributed to detrimental effects on spermatogenesis, due to the undescending testis which ultimately leads to infertility as already reported (21, 35–37).

In the present study, OX2R binding to OXB missed to play a direct steroidogenic role in the testis, in contrast a steroidogenic activity occurs when OXA binds to OX1R (6, 9). Thus, the physiological role of OXB in Leydig cells remains unclear. In fact, OXB might regulate other Leydig cell activities or act on other testicular cells through a paracrine manner (15, 16).

In normal and cryptorchid epididymides, OXB-IR has been found in different compartments along the entire length of the organ, while OX2R-IR has been only found in the caput of normal and cryptorchid animals.

PPO and OX2R expressions in the testis and epididymis—both in normal and pathological conditions—was ascertained by Western Blotting. *PPO* expression has already been reported in testis from normal and cryptorchid dogs (9) previously, as well as in the South American Camelid alpaca testis (8). In the present study, *OX1R* mRNA expression levels were evaluated in all tissue groups in order to better clarify the mechanism of action of the entire orexinergic complex. The presence of *PPO* and *OX2R* in different tracts of normal and cryptorchid epididymis supports the hypothesis that *OXB* might be derive from the precursor molecule PPO. However, these results are not completely in accordance with the immunohistochemical results, by the latter, OX2R was absent in the corpus and cauda of both the normal and cryptorchid epididymis. This discrepancy in results might be ascribed to: (a) higher sensitivity of the molecular protocols compared to immunohistochemistry; (b) high turnover of cellular production/internalization of orexins. Finally, in this study, the *in vitro* tests demonstrated no steroidogenic effects promoted by OXB in all tested groups. This result is in agreement with previous findings which show that OXB altered neither T nor 17βE productions in normal rat testis (15). In contrast, OXA-binding OX1R promoted T stimulation in rats (5), alpaca (8), mice (11, 13), and normal and cryptorchid canine (9) testes. Specifically, in the testis from normal and cryptorchid dogs, it has been shown that the increase of T production, OXA-mediated and the subsequent decrease in 17βE biosynthesis was modulated by OXA-evoked ARO activity inhibition.

Considering the null effect exerted by OXB-binding OX2R on steroidogenesis, which the role of this peptide in the regulation of testicular and epididymal functions? The most interesting points of discussion of this work are summarized as follow: (a) mRNA expression levels of all three genes reduced from the testis to the cauda epididymis in normal animals, and increased in each segment of the cryptorchid dogs when compared with the normal ones; (b) the increase in the *OX2R* mRNA level was particularly high in the testis and in the caput epididymis of cryptorchid dogs; (c) an increased *OX1R* mRNA level was particularly detected in the corpus of cryptorchid epididymis. The high level of *PPO* could be ascribed to the simultaneous presence of OXA and OXB peptides. As reported previously, OXB and OX2R were described in the interstitium and in the seminifeous tubules, as well, of canine testis. Although, OXB was demonstrated to not be involved in the testicular steroidogenesis, its localization in many tubular cytotypes (present work and our previous papers) led us to hypothesize a possible implication in spermatogenesis regulation (15, 16). The supranuclear localization of OXA and OXB in the principal cells of the epididymis demonstrated that these molecules might locate at different sites, suggesting that the mode of action might be paracrine. Studies by Crabo et al. (38) have shown that the proximal region of the epididymis and the epididymal efferent ductules are involved in absorbing 90% of the fluids secreted by the seminiferous tubules. The cytoplasmic localization of OXB and OX2R in the caput epididymis and in the efferent ductules led us to hypothesize that the fluid produced in the testis was re-absorbed by the efferent ductules, and by the proximal portion of the epididymis. Therefore, it was speculated that OXB-binding OX2R might play a role in the absorption and secretion process at the epididymis level, highlighted more in the epididymal proximal portions.

Cryptorchidism is established as a risk element in the determinism of infertility and testicular germ cell tumors in men (22). Although the clear mechanism is poorly known, the depletion of germ cells and apoptotic events were the consequence of elevated testicular temperature in the abdominal retained gonad. Later stage haploid germ cells seemed to be the most liable to high temperatures (22). Leydig and Sertoli cells are relatively resistant to the thermal effect of cryptorchidism; instead, they undergo hyperproliferation. Undescended testis is characterized by spermatogonial disruption, which sometimes might be the reason of abnormal germ cell differentiation and the formation of testicular germ cell tumors. Nonetheless, in physiological state, orexins may act in enhancing cell proliferation and survival (39). Orexins, by binding OX1R or OX2R, can cause massive apoptosis and a reduction in cell growth in several cancer cell lines, such as human colon cancer cells (40, 41), human neuroblastoma cells (40), rat pancreatic tumor cells (42), rat C6 glioma cells (43), and Chinese hamster ovary (CHO) cells transfected with *OX1R* cDNA. This apoptosis-induced mechanism by orexins seems to be related to the discharge of cytochrome c from mitochondria and the activation of caspase-3/7 OX1R-mediated (41). Moreover, OXA can induce apoptosis *via* OX2R in rat pancreatic tumor cells and in rat C6 glioma cells (42, 43). On this basis, it was speculated that elevated mRNA *PPO* and orexin receptors levels in cryptorchid organs might be evoked by the orexinergic complex, since pre-neoplastic transformation has been demonstrated to be associated to the induction of *OX1R* and/or *OX2R* expression and the subsequent the activation of orexin receptor-evoked apoptosis.

Autophagy is defined as the initial phenomenon in hyperthermic conditions associated with testicular spermatogenesis damage (44, 45). Previous findings have demonstrated that OXA generated autophagy through the ERK pathway in HCT-116 human colon cancer cells. Intriguingly, apoptosis and autophagy were described as synchronous phenomena involving in promoting testicular germ cell death. Cryptorchidism is also associated with epididymal anomalies (46, 47), with aberrations in mitochondrial structure. Sperm mitochondria produce reactive oxygen species (ROS), which can determine damages of an oxidative nature (48, 49). In particular, ROS stimulated autophagy *in vitro* (50) and apoptosis-like phenomenon in male gametes (48, 49). A correlation between OXB and its control on redox status was described in swine ovarian follicles too (51). Based on such evidence, the high level of *PPO* and *OX2R* genes found in the testis and caput epididymis led us to hypothesize that *OXB* binding *OX2R*, might regulate the redox status inducing autophagy in the testis and a pro-apoptotic effect in the caput epididymis; in addition, an increased expression level of Heath shock protein-70 (HSP 70) transcript was found in human cryptorchid corpus and cauda epididymis, as well as in vas deferens (52). Further studies are necessary to better clarify the before mentioned hypotheses.

## Conclusions

This study corroborates the evidence of a different distribution of OXB and OX2R in the testis and epididymis of normal and cryptorchid dogs. These peptides in healthy dogs are hypothesized to be involved in the regulation of testicular spermatogenesis and epididymal absorptive and secreting activities. On the basis of our results, and at the light of what is known in literature, it could hypothesized that OXB- binding OX2R, may activate autophagy in the testis and/or cause a pro-apoptotic effect in the caput epididymis by regulating the redox status, respectively; as well as OXA-binding OX1R might modulate HSP 70 expression at the level of corpus epididymis. These results represent the basis for further functional and molecular studies addressing the possible roles of OXB and OX2R in normal and pathological conditions of the male reproductive system.

## Data Availability Statement

The raw data supporting the conclusions of this article will be made available by the authors, without undue reservation.

## Ethics Statement

All experimental procedures were approved by the Ethical Animal Care and Use Committee of the University of Naples Federico II, Department of Veterinary Medicine and Animal Production, Naples, Italy (no. 0,050,377). Written informed consent was obtained from the owners for the participation of their dogs in this study.

## Author Contributions

CS, AP, NM, and GL contributed to the design and implementation of the research, interpretation of results, and to the critical review of the manuscript draft. The *in vitro* experiments were collected by LA. AC and LV provided assistance for analyzing the data. The paper was revised and agreed by CS, AP, LA, AC, LV, NM, and GL. All authors contributed to the article and approved the submitted version.

## Conflict of Interest

The authors declare that the research was conducted in the absence of any commercial or financial relationships that could be construed as a potential conflict of interest.

## Publisher's Note

All claims expressed in this article are solely those of the authors and do not necessarily represent those of their affiliated organizations, or those of the publisher, the editors and the reviewers. Any product that may be evaluated in this article, or claim that may be made by its manufacturer, is not guaranteed or endorsed by the publisher.
