# Supplementary figures and images for: Does Orexin B-Binding Receptor 2 for Orexins Regulate Testicular and Epididymal Functions in Normal and Cryptorchid Dogs?
Source: Front Vet Sci. 2022 Jul 12;9:880022. doi: 10.3389/fvets.2022.880022 (PMC9323089; doi:10.3389/fvets.2022.880022)

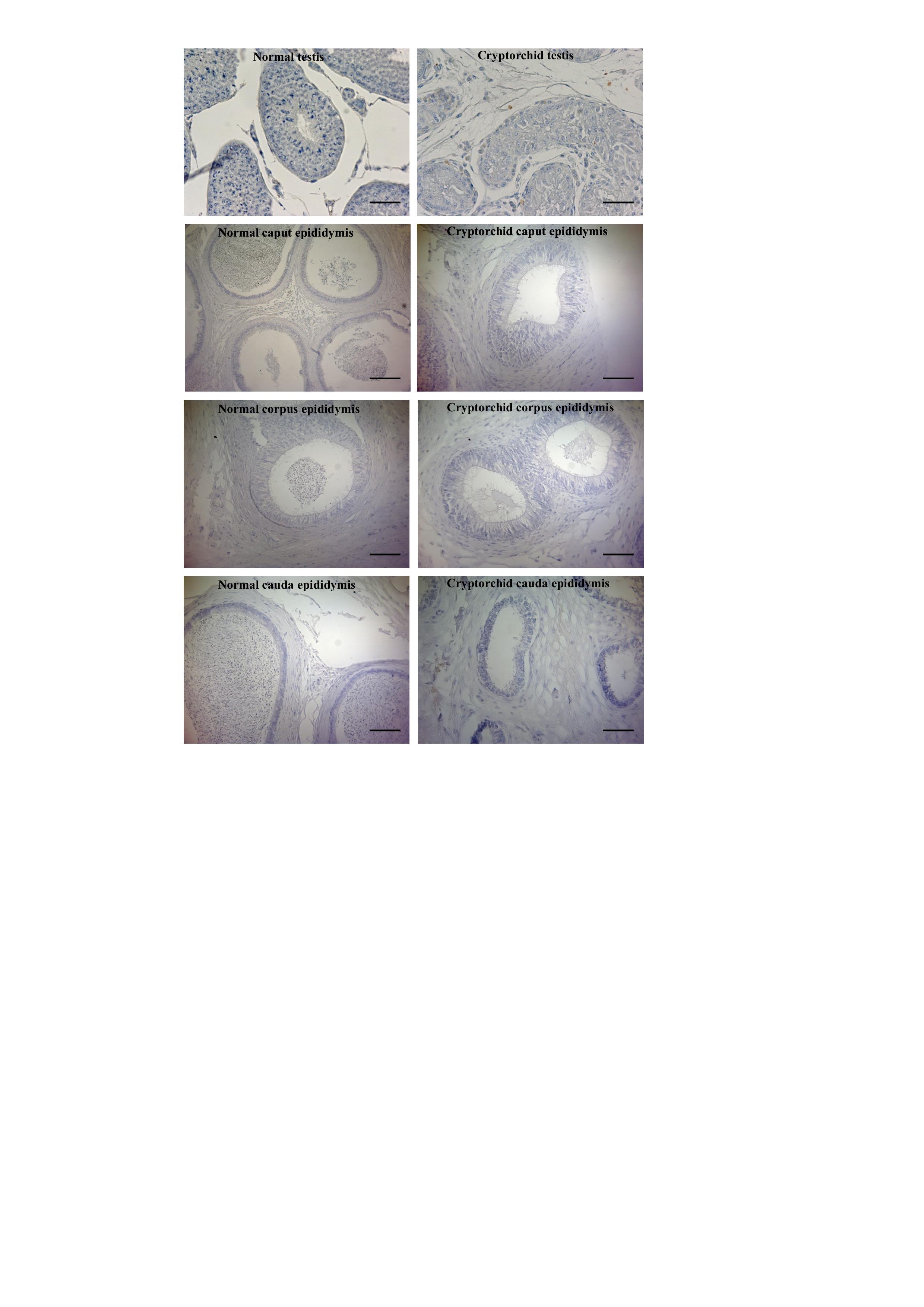

Supplement: Supplementary file 1 [file Image_1.TIFF]

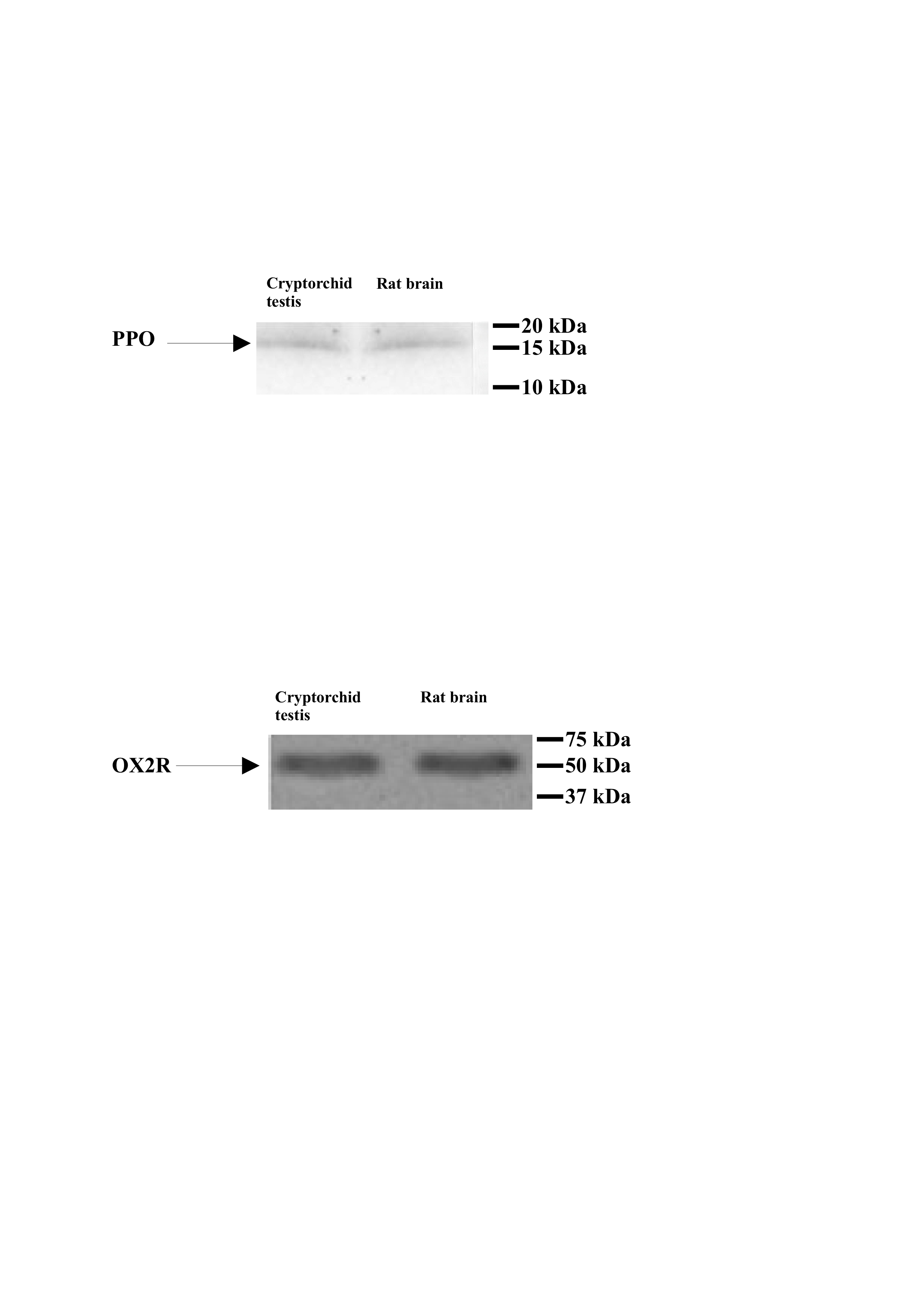

Supplement: Supplementary file 2 [file Image_2.TIFF]
